# Morbidity and Mortality Outcomes of Cytoreductive Surgery and Hyperthermic Intraperitoneal Chemotherapy at a Single Institution in Japan

**DOI:** 10.1155/2012/836425

**Published:** 2012-06-18

**Authors:** Akiyoshi Mizumoto, Emel Canbay, Masamitsu Hirano, Nobuyuki Takao, Takayuki Matsuda, Masumi Ichinose, Yutaka Yonemura

**Affiliations:** ^1^Department of Surgery, Kusatsu General Hospital, Yabase Kusatsu 1660, Japan; ^2^General Surgery Clinic, Kocaeli Derince Education and Research Hospital, Kocaeli, Turkey; ^3^NPO Organization to Support Peritoneal Dissemination Treatment, Osaka, Japan

## Abstract

*Background*. Even though cytoreductive surgery (CRS) and hyperthermic intraperitoneal chemotherapy (HIPEC) are associated with a high morbidity and mortality rates, it has been reported that CRS and HIPEC improved survival of selected patients with peritoneal carcinomatosis. We aimed to report morbidity and mortality results of CRS and HIPEC from a single institution in Japan. *Methods and Results*. Total of 284 procedures of CRS were performed on patients with pseudomyxoma peritonei, peritoneal carcinomatosis (PC) from colon cancer and gastric cancer between 2007 and 2011 in our institution. The morbidity rate was 49% of all procedure, and grades I/II and grades III/IV complications were 28% and 17%, respectively. Most frequent complication was surgical site infections including intraabdominal abscess. The mortality rate was 3.5%, and reoperation was needed in 11% of all procedures. Univariate and multivariate analysis showed peritoneal carcinomatosis index (PCI) greater than 20 was the only significant factor for occurrence of postoperative complications (*P* < 0.01). In contrast, HIPEC significantly reduced postoperative complications (*P* < 0.05). *Conclusions*. The morbidity and mortality rates of our institution are comparable with previous reports that are in acceptable rates. Optimal patient selection such as patients with PCI less than 20 seems to be of paramount importance to CRS and HIPEC.

## 1. Introduction

Peritoneal carcinomatosis (PC) originated from gastrointestinal tract malignancies has been regarded as a lethal condition, and these patients have considered to receive systemic chemotherapy or palliative therapy. However, long-term survival is difficult to obtain by systemic chemotherapy. Sugarbaker [1] developed a novel therapeutic approach in the treatment of peritoneal surface malignancies with combination of peritonectomy and intraperitoneal chemotherapy applications in the mid 1990s. Since then, cytoreductive surgery (CRS) and hyperthermic intraperitoneal chemotherapy (HIPEC) have been recognized as a useful treatment for patients with PC arising from gastrointestinal cancer, gynecological malignancies or primary peritoneal surfaces malignancies as mesothelioma. Although survival benefit of this procedure has been reported in numerous literatures, this treatment is still not widely accepted worldwide because of the necessity of long learning curves for application of these techniques and high postoperative mortality and morbidity rates.

Literatures concerning CRS in Japan are almost limited to the gynecological field. Postoperative complication after CRS and HIPEC for PC originated from gastrointestinal malignancies has not been reported in Japan, except for PC originated from gastric cancer [2]. The purpose of this study is to investigate the morbidity and mortality outcomes of CRS and HIPEC for patients with pseudomyxoma peritonei and PC originated from colon cancer and gastric cancer in the single institution of Japan.

## 2. Methods

### 2.1. Patients

Patients treated at the Kusatsu General Hospital between 2007 and 2011 with a diagnosis of PC were included in this study. Patients with extraperitoneal lesions were excluded from the study by contrast-enhanced computed tomography (CT) scans and/or positron emission tomography (PET). Careful preoperative evaluations including physical examination, hematological laboratory data, and cardiopulmonary function were performed. Patients characteristic are shown in Table 1. Total of 284 procedures of CRS were performed on patients with PC that those of 236 procedures on 205 patients were with pseudomyxoma peritonei, 32 procedures with 29 patients with PC originated from colon cancer, 16 procedures on 16 patients with PC originated from gastric cancer. They were 78 males (31%) and 172 females (69%). The mean ± SD age was 57 ± 13 years (range from 23 to 88 years old). This study was approved by the Local Ethical Committee of Kusatsu General Hospital.

### 2.2. Cytoreductive Surgery (CRS)

All procedures were performed by the same surgical team, led by a single surgeon (Y. Yonemura). A mid-line skin incision from xiphoid process to pubic tubercle was performed. Peritoneal carcinomatosis index (PCI) was evaluated at the time of laparotomy as described before [3]. CRS included several visceral resections such as stomach, colon, ovary, uterus, spleen, gallbladder, and small bowel. Parietal peritonectomy, greater omentectomy, and lesser omentectomy were also included. The residual tumors were classified intraoperatively using the completeness of cytoreduction (CC) score [4]. CC-0 indicates that no macroscopical tumors remained and CC-1 residual tumor nodules less than 2.5 mm. CC-2 and CC-3 indicate residual tumor nodules between 2.5 mm and 2.5 cm and >2.5 cm, respectively.

### 2.3. Hyperthermic Intraperitoneal Chemotherapy (HIPEC)

CRS was followed by HIPEC. Two inflow and one outflow drainage tubes were placed subphrenically and in the pelvic cavity, respectively. Abdominal cavity was lavaged 10 times by 1 L of normal saline before HIPEC. Then, heated normal saline was circulated for 60 minutes by using a roller pump and heat exchanger. 20 mg of mitomycin C and 100 mg of cisplatin were used as chemotherapeutic agents. Intraperitoneal temperature was monitored by placement of a thermometer in the abdominal cavity and maintained at approximately at 41-42°C. After HIPEC, abdominal cavity was lavaged by 10 times of 1 L of normal saline. HIPEC were not performed if a high risk of postoperative complications was concerned. Therefore, patients with poor preoperative performance status, serious laboratory data, intraoperative excessive bleeding, and very aggressive operation procedures did not receive HIPEC.

### 2.4. Data and Statistical Analysis

Data expressed the mean ± standard deviation, when appropriate. Postoperative complication was assessed based on Common Terminology Criteria for Adverse Events v 4.0. Content of postoperative complications was determined by the first complication observed after surgery or was selected more severe complication when multiple complications occurred almost simultaneously. All analyses were performed using StatMate IV for Windows (Atms, Tokyo, Japan). ANOVA, chi-square test or Fisher exact test was used to compare the number of postoperative complications when appropriate. Multivariate analysis was performed using a logistic regression analysis to detect independent risk factors for postoperative complications. *P* < 0.05 was considered as significant.

## 3. Results

Details of patients characteristic are shown in Table 1. The mean age of patients with gastric cancer was significantly younger than that of the patients with pseudomyxoma peritonei or colon cancer (*P* < 0.01). The mean PCI ± SD was 20 ± 13 in all patients, and was 22 ± 12 in pseudomyxoma peritonei, was 12 ± 11 in colon cancer, and was 10 ± 10 in gastric cancer, respectively. The mean PCI of pseudomyxoma peritonei was significantly higher than that of colon cancer or gastric cancer (*P* < 0.01). The mean operating time was 288 ± 96 minutes, and there were no significant difference among patients groups. The mean intraoperative blood loss was 2.4 ± 2.1 L in all procedures, and that of pseudomyxoma peritonei was significantly higher than that of colon cancer or gastric cancer (*P* < 0.01).

Of the 284 CRS for PC, 64% of all procedures underwent to CC-0 or CC-1 resection, whereas 36% of all procedures resulted in CC-2 or CC-3 resection. There were no significant differences among pseudomyxoma peritonei, colon cancer and gastric cancer group, in terms of completeness of cytoreduction. HIPEC was performed in 64% of all procedures.

### 3.1. Morbidity and Mortality

The morbidity rate was 49% (139/284) in all procedures (Table 1). According to Common Terminology Criteria for Adverse Events, 80 cases (28%) were associated with grades I/II complications and 49 cases (17%) with grades III/IV complications in all procedures. grades III/IV complications in gastric cancer group (38%) seemed to be higher than that in the other groups, but the difference was not statistically significant.

Most frequent complication was surgical site infections including intraabdominal abscess, which was 46% (64/139) of total number of postoperative complications (Table 2). Gastric or small intestinal perforation, postoperative ileus, anastomotic leakage, urinary disturbance, intestinal fistula and postoperative bleeding were the other main complications after cytoreductive surgery and HIPEC. Gastric or small intestinal perforation, intraabdominal abscess, anastomotic leakage, and postoperative bleeding were the main severe complications as grade III complications.

Postoperative death within 30 days was observed in 10 cases (3.5%). The mortality rate was 3.8% (9/236) in pseudomyxoma peritonei group, 3% (1/32) in colon cancer group, and none in gastric cancer group (Table 1). The causes of death were anastomotic leakage, intestinal fistula, postoperative bleeding, sepsis, and DIC. Sepsis or multiorgan failure was developed due to anastomotic leakage or intestinal fistula. Reoperations were needed in 11% of all procedures (32/284). In particular, all cases of postoperative bleeding, and most cases of gastric or intestinal perforation were required reoperation (Table 2).

### 3.2. Learning Curve

When divided into two groups; the first 142 procedures and the latter 142 procedures, postoperative complication rate was 49% (69/142) and 47% (67/142), respectively. The occurrence of grades I/II, grades III/IV and grade V complication in the first half were 27% (38/142), 18% (26/142), and 3.5% (5/142), respectively, and those in the latter half were 27% (39/142), 16% (23/142) and 3.5% (5/142), respectively. There was no significant difference between groups (*P* > 0.05).

### 3.3. Risk Factors Associated with Postoperative Complications

Univariate analysis showed that PCI greater than 20, operation time longer than 5 hours, and blood loss greater than 2.5 L were the significant risk factors for the occurrences of postoperative complications. On the other hand, the complication rate in patients received HIPEC was significantly lower than that in the patients without HIPEC. Gender, age divided into 65 years old, origin of peritoneal carcinomatosis, or completeness of cytoreduction were not related to the occurrence of postoperative complications (Table 3).

Multivariate analysis using a logistic regression model showed that PCI higher than 20 was the only significant factor which increased the occurrence of postoperative complications. PCI greater than 20 was associated with 2.8 times increased the risk of the occurrence of postoperative complications (Table 4). Patients who receive HIPEC showed significant lower mortality and morbidity rate than patients not received HIPEC after multivariate analysis.

## 4. Discussion

PC of gastrointestinal origin has been regarded as inoperable conditions and treated by systemic chemotherapy or palliative therapy. Based on the theory that peritoneal carcinomatosis is a locoregional disease, cytoreductive surgery and perioperative intraperitoneal chemotherapy have been applied in selected patients with peritoneal carcinomatosis. This procedure has achieved a 5-year survival rate of 73% in patients with pseudomyxoma peritonei [3], 45% in patients with PC of colon cancers [5], and 27% in patients with PC of gastric cancer [2]. However, high morbidity and mortality rates remain a serious concern of cytoreductive surgery and HIPEC.

Chua et al. [6] reviewed that a major morbidity rates ranges from 12% to 52% in high-volume centers. In the present study, the morbidity rate of 49% in all procedures and 21% more than grade III complication were observed, which were within the reported ranges. Considering severe situation of patients with PC and aggressive surgical method of cytoreductive surgery, the morbidity rate is thought to be acceptable because of obtained survival benefit from these procedures.

The most frequent complication in our institution was surgical site infections including intra-abdominal abscess, which occupied 46% of all complications. Intra-abdominal abscess was diagnosed by dirty discharge from drainage tube and computed tomography. Surgical site infections including intraabdominal abscess could be treated usually by drainage of infected site (81%, 52/64), by needle puncture to abscess cavity using ultrasound device or computed tomography (13%, 8/64), or by surgical reoperation (6%, 4/64). Surgery-related complications such as gastric or intestinal perforation, anastomotic leakage, intestinal fistula, and postoperative bleeding were other major complications after cytoreductive surgery and HIPEC.

The mortality rate after cytoreductive surgery and HIPEC has been reported to be ranging from 0.9% to 5.8% [6]. A mortality rate in our institution was 3.5% in all procedures. The cause of death included anastomotic leakage, sepsis, postoperative bleeding, intestinal fistula, and DIC. Reoperation was needed in 11% of all procedures. Serious complications required reoperations were gastric or intestinal perforation and postoperative bleeding.

Risk factors associated with postoperative complications from univariate analysis were PCI greater than 20, duration of operation longer than 5 hours and intraoperative bleeding greater than 2.5 L. Multivariate analysis showed that only PCI >20 was the significant risk factor for the occurrence of postoperative complications. Chua et al. [7] showed left upper quadrant peritonectomy and small bowel resection were the factors that predicted for a poor perioperative outcome. Saxena et al. [8] showed that ASA more than 3 and an operation length more than 10 h were the significant risk factors for grades IV/V morbidity in patients with pseudomyxoma peritonei.

It has been demonstrated that HIPEC is not a significant risk factor associated with postoperative complications, although HIPEC may affect bone marrow activity, renal function or operating duration. Unexpectedly, we found that patients received HIPEC were associated with significant lower complication rate by univariate and multivariate analysis. The reason is not known, but the indication of which we usually did not apply HIPEC on patients with CC-2/3, excessive intraoperative bleeding, poor laboratory data or performance status more than 3, might affect the results obtained in this study. Further studies are needed to clarify the role of HIPEC in postoperative complications after CRS.

Elias et al. [3] and Glehen et al. [9] reported that a risk of morbidity and mortality after cytoreductive surgery and HIPEC significantly is related to the institution, where the treatment was performed and concluded that this procedure should be centralized to institutions with expertise in the management of peritoneal carcinomatosis. Moreover, it is demonstrated that learning curve is an important factor to reduce the occurrence of postoperative complications [10, 11]. Approximately, 130–140 cases are reported to be necessary to minimize mortality and morbidity after the procedure [10, 11]. In this study, we did not find any difference between the first 142 and the latter 142 cases in terms of the occurrence of postoperative complications. The significant difference between groups was the ratio of CC-0/1 and CC-2/3 resections. CC-0/1 resection was performed 55% of the procedures (78/141) in the first half and 77% (108/141) in the latter half (*P* < 0.05, data not shown). Regarding the similar complication rates between two groups, we thought that we performed all surgical procedures with one experienced surgeon. Accumulation of experience in this procedure in our experienced surgeon could be the reason similar complication rates in the two groups. Even though, morbidity and mortality rates were not differed between two groups, complete resection rates were increased in latter group still suggesting that there was a learning curve associated with this procedure.

In conclusion, the morbidity and mortality rate after cytoreductive surgery and HIPEC in our institution did not differ from previous reports. The morbidity and mortality rate after the procedure are compatible to those after pancreaticoduodenectomy or esophagotomy, which are widely performed all over the world. Up to now, long-term survival of patients with PC can be obtained only by cytoreductive surgery and HIPEC. This procedure could be, therefore, considered as a standard treatment of PC in selected patients.

## Figures and Tables

**Table 1:** Characteristics of the patients with peritoneal carcinomatosis.

Diagnosis	Pseudomyxoma peritonei	Colon cancer	Gastric cancer	All
Number of patients	205	29	16	250
Number of operations	236	32	16	284
Gender (male/female)	61/144	12/17	5/11	78/172
Age	58 ± 13 (28–88)	54 ± 14 (23–78)	48 ± 13 (30–68)	57 ± 13 (23–88)
PCI score	22 ± 12 (0–39)	12 ± 11 (0–39)	10 ± 10 (0–30)	20 ± 13 (0–39)
Operating time (minutes)	292 ± 100 (30–535)	257 ± 71 (95–413)	275 ± 67 (182–384)	288 ± 96 (30–535)
Blood loss (L)	2.6 ± 2.2 (0.5–11)	1.4 ± 1.2 (0.4–6.5)	1.9 ± 1.2 (0.5–4.5)	2.4 ± 2.1 (0.5–11)
CC 0, 1/CC 2, 3	147/89 (62%/38%)	25/7 (78%/22%)	11/5 (69%/31%)	183/101 (64%/36%)
HIPEC (yes/no)	141/95 (60%/40%)	27/5 (84%/16%)	13/3 (81%/19%)	181/103 (64%/36%)
Complications	118 (50%)	12 (38%)	9 (56%)	139 (49%)
None	118 (50%)	20 (68%)	7 (44%)	145 (51%)
Grades I/II	72 (31%)	5 (16%)	3 (19%)	80 (28%)
Grades III/IV	37 (16 %)	6 (19%)	6 (38%)	49 (17%)
Grade V	9 (3.8%)	1 (3%)	0	10 (3.5%)

**Table 2:** Morbidity and mortality after cytoreductive surgery.

	Grades I/II	Grades III/IV	Grade V	All	Reoperation
SSIs; intraabdominal abscess	52	12		64	4
Gastric or intestinal perforation	2	13		15	11
Postoperative ileus	9	5		14	2
Anastomotic leakage	3	6	3	12	4
Urinary disturbance	9	1		10	
Intestinal fistula	5	3	1	9	2
Postoperative bleeding		6	2	8	8
Sepsis			3	3	
DIC		1	1	2	
Respiratory distress		1		1	
Diaphragmatic hernia		1		1	1

Total	80	49	10	139	32

SSIs: surgical site infections.

**Table 3:** Univariate analysis of variables associated with postoperative complications.

Variables	Complications	No complications	*P*
Gender			0.47
Male	44	42	
Female	92	106	

Age			0.84
<65	98	105	
>65	38	43	

Diagnosis			0.39
Pseudomyxoma peritonei	115	121	
Colon cancer	12	20	
Gastric cancer	9	7	

PCI			<0.001
<20	48	94	
>20	85	51	

Operation time (hr)			<0.05
<5	63	91	
>5	73	57	

Blood loss (L)			<0.001
<2.5	67	113	
>2.5	68	35	

Completeness of cytoreduction			0.13
CC-0/1	81	102	
CC-2/3	55	46	

HIPEC			<0.001
Yes	72	109	
No	64	39	

**Table 4:** Multivariate analysis of risk factors for postoperative complication.

Variable	Hazard ratio	95% CI	*P *value
PCI	2.83	1.46–5.49	<0.01
Operation time	1.79	0.97–3.29	0.06
Blood loss	1.69	0.94–3.05	0.08
HIPEC	0.34	0.16–0.69	<0.01
